# The Influence of Oil and Thermal Aging on the Sealing Characteristics of NBR Seals

**DOI:** 10.3390/polym16172501

**Published:** 2024-09-02

**Authors:** Yiding Li, Jian Wu, Zhihao Chen, Ziqi Zhang, Benlong Su, Youshan Wang

**Affiliations:** 1 Center for Rubber Composite Materials and Structures, Harbin Institute of Technology, Weihai 264209, China; 2 National Key Laboratory of Science and Technology on Advanced Composites in Special Environments, Harbin Institute of Technology, Harbin 150090, China

**Keywords:** nitrile butadiene rubber (NBR), aging behavior, seal, contact pressure, surface morphology, sealing characteristics

## Abstract

Nitrile Butadiene Rubber (NBR) is widely used as a sealing material due to its excellent mechanical properties and good oil resistance. However, when using NBR material, the seal structure is unable to avoid the negative effects of rubber aging. Hence, the influence of oil and thermal aging on the characteristics of NBR seals was studied by coupling the mechanical behavioral changes with the tribological behavioral changes of NBR in oil and the thermal environment. For this paper, aging testing and compression testing of NBR were carried out. Additionally, friction testing between friction pairs under different aging times was carried out. The surface morphology of the NBR working surface under different aging conditions was also observed. Finally, coefficients of different test conditions were introduced into the finite element model of NBR seals. It can be seen from the results that the elastic modulus increased with the increase in aging time in the thermal oxidative aging testing. The elastic modulus after 7 days of thermal oxidative aging increased by 135.45% compared to the unaged case, and the elastic modulus after 7 days of oil aging increased by 15.03% compared to the unaged case. The compression set rate of NBR increased significantly with the increase in aging time and temperature. The coefficient of friction (COF) between friction pairs increased first and then decreased with the increase in aging time. The maximum contact pressure decreased by 2.43% between the shaft and sealing ring and decreased by 4.01% between the O-ring and groove. The proportion of the effective sealing area decreased by 3.05% between the shaft and sealing ring and decreased by 6.11% between the O-ring and groove. Furthermore, the sealing characteristics between the O-ring and groove were better than those between the shaft and sealing ring.

## 1. Introduction

Nitrile butadiene rubber (NBR) is widely used in sealing structures due to its excellent mechanical properties, good oil resistance and good oil impermeability [1,2]. In general, NBR seals are always exposed to high ambient temperatures and they are always kept under load in oil environments. The mechanical properties and tribological behaviors of NBR usually change due to different factors, such as the aging and swelling [3,4,5] of the rubber. They result in the change of the sealing characteristics [6,7,8]. Therefore, it is necessary and important to study the influence of oil and thermal oxidation aging on the sealing characteristics of NBR seals.

In recent years, some researchers have been focused on the environmental factors affecting rubber aging, such as the working temperature and the UV intensity. The related aging mechanism [9,10,11,12,13] has also been studied. A. Mostafa et al. [14] carried out a thermal aging test in different temperature ranges. The effects of aging temperatures on the tension, compression and hardness properties of styrene butadiene rubber (SBR) and nitrile butadiene rubber (NBR) were investigated. The dependence of mechanical properties on the aging temperature and carbon black (CB) loading [15] were obtained through a combination of theoretical analysis and experimental verification. Young Seok Lee et al. [16] studied the thermal characteristics and thermal aging properties of an acrylonitrile butadiene rubber composite in relation to acrylonitrile (ACN) content. It could be seen from the results that the elongation at break and elastic recovery [17] of NBR were decreased by subjecting the composite to thermal-oxidative and thermal aging processes. Nur Aini Safiah Abdullah et al. [12] observed the effect of temperature on the mechanical properties of a selected elastomer. The conclusion that the temperature was one of the aging factors of the elastomer was proved. The effects of ambient humidity [18,19,20] and UV intensity [21,22,23] on rubber aging were also studied by some research. For example, nitrile rubber is susceptible to moisture absorption and hydrolysis in high-humidity environments and these factors decrease the life of rubber seals. Microorganisms easily reproduce in a high-humidity environment, leading to the decomposition of some components of rubber, thus accelerating the aging of rubber. Therefore, it is important to establish the testing conditions of NBR according to its service conditions to observe specific aging behaviors and the mechanical characteristics of NBR.

Surface quality [24,25,26] is important to NBR seals, especially the type of surface material and the roughness of the sealing surface. The contact area and tightness of the seal can be affected by the surface roughness. Rough and uneven surfaces tend to produce small contact gaps [27], leading to media leakage [28,29,30,31] and reduced sealing performance. Some researchers studied the working surface of seals. A. Y. Jiao et al. [32] created a magnetic abrasive finishing (MAF) process. The problem that traditional finishing methods encountered was that they were difficult to implement on the bottom and sides, but synchronization of finishing resolved this issue. Fu-Ying Zhang et al. [33] calculated and analyzed the influence of structural parameters on pumping rate. The macroscopic pumping effect model of the rotary shaft lip seal and the pumping rate equation were developed and obtained. The main factors that significantly influenced the seal performance were found and they were a low angle, a high angle and interference. Zheng-peng Gu et al. [34] built an experimental apparatus for leakage quantization and provided general guidance for the design of parameters of static seals [35,36,37]. The coefficient of friction (COF) and the amount of wear can be used to characterize the lubricating properties of the seal. A low COF reduces friction between friction components, thereby reducing wear and heat and increasing the service life of the seal. To improve the characteristics of the sealing, some researchers have been dedicated to studying the methods and critical factors of different conditions. Avanzini A. [38] carried out the testing of four thermoplastic materials: polyamide 6 (PA 6) [39], thermoplastic polyurethane (TPU) [40] and ultra-high-molecular-weight polyethylene (UHMWPE) [41] reinforced with glass or ceramic microspheres to enhance the wear resistance of seals. Orcun Saf et al. [42] proposed an alternative method for elastic characterization utilizing impedance tube-based sound transmission loss (STL) measurements. A wafer-level sealing characterization method [43] using the pressure dependence of the mechanical quality factor of Si micro cantilevers has also been developed. It is necessary to combine the influence of critical factors in actual service conditions when investigating sealing characteristics.

This study focused on the variation of sealing characteristics using finite element modeling and simulation analysis. The mechanical behavioral changes were coupled with the tribological behavioral changes of NBR in oil and thermal environment. An experimental process combining aging testing, compression set testing and compression testing was adopted. Subsequently, the two-dimensional friction test bench was used to carry out friction testing to obtain the tribological behavior laws of sealing friction pairs under actual sealing conditions. ABAQUS 6.12 software was used for modeling and simulation analysis. The effects of different aging testing conditions (oil aging and thermal oxidative aging) on both the mechanical properties and the tribological behaviors of NBR were studied by analysis of testing results and surface morphology. The influence of aging on the magnitude and distribution of the contact pressure between sealing friction pairs was also focused on and observed.

## 2. Materials and Methods

### 2.1. Materials

Experimental rubber specimens were prepared by vulcanization. The manufacturing company was Caddy Northwest Rubber Company in Xianyang City, China. GJB 250A–96 [44] was selected as the material standard. The type of NBR material used in the paper was 5171. The batch production of the NBR material used in the paper was 2023–38#. According to the rubber product card, the recommended vulcanization time was 40 minutes (2400 s) and the recommended temperature was 151 °C.

**Figure 1:**
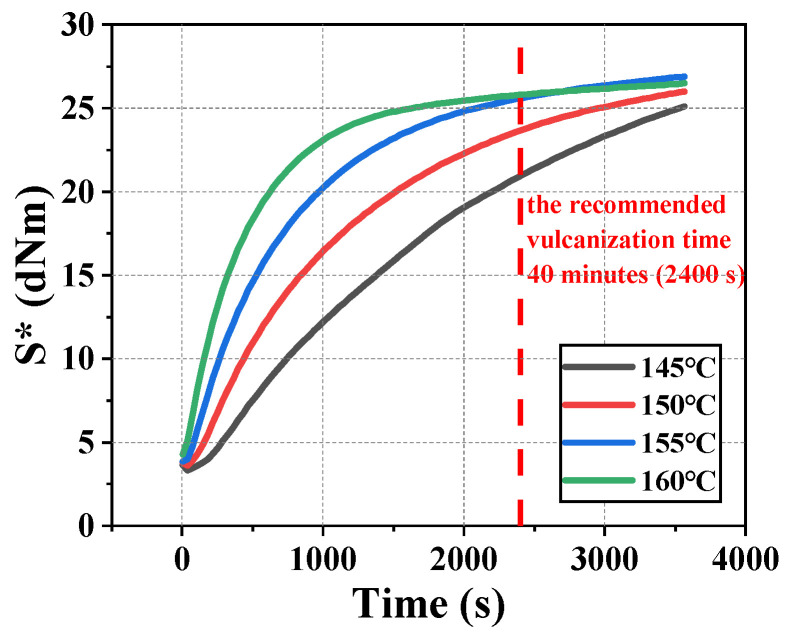
Evolution of S* with vulcanization time at four different vulcanization temperatures (The red dotted line in the figure refers to the recommended vulcanization time).

In this experiment, the rubber vulcanization analyzer (RPA S5, Songshu Testing Instruments Company, Dongguan, China) was used to obtain the appropriate temperature. Four vulcanization temperatures (145 °C, 150 °C, 155 °C and 160 °C) which were close to 151 °C were selected for the pre-vulcanization experiments. Mechanical information curves are exported by a vulcanization analyzer. Under the service conditions of NBR seals, seals are in the state of oil film friction with the metal structure. Shear strain near the working surface of the sealing friction pairs is always large. Therefore, S* was chosen to characterize the shear property of rubber material, as shown in Figure 1. It can be observed from Figure 1 that the temperature range from 155 °C to 160 °C is more appropriate than the recommended temperature (151 °C) when the time is 40 minutes (2400 s in Figure 1). The shear properties of NBR vulcanized at 155 °C are better than those at 151 °C. Therefore, the vulcanization temperature was set to 155 °C and vulcanization time was set to 40 minutes.

Standard specimens were prepared using a vulcanizing machine under a 15 MPa working pressure. Bubbles in rubber were removed by three rounds of pressurization and depressurization processes before formal vulcanization to improve the final quality of standard specimens. NBR cylindrical specimens (diameter is 10.0 ± 0.2 mm, height is 10.0 ± 0.2 mm) were obtained and they were used for aging testing and subsequent compression testing.

### 2.2. Aging Testing

**Figure 2:**
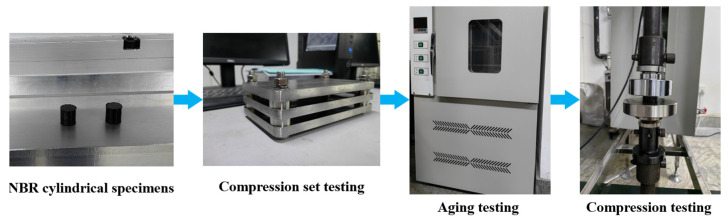
The experimental procedures for NBR specimens, including compression set testing, aging testing in thermal oil and thermal air environment and compression testing.

In accordance with the service conditions of the combined sealing structure, the thermal oxidative environment and oil environment were set up for the tests. GB/T 3512–2014 [45] and GB/T 1690–2010 [46] were selected as testing standards. And the aging test chamber (401-A, Yangzhou Daochun Testing Machinery Factory, Yangzhou, China) was selected. The aging tests lasted a total of 7 days and the aging temperature was set to 125 °C according to the above testing standards. Figure 2 shows the testing procedures of NBR specimens. The compression fixture was used to consider the compression set behavior of NBR materials and simulate the loaded state of NBR seals. The required compression rate was determined as 20% according to GB/T 7759.1–2015 [47]. The aging tests were carried out using cylindrical specimens under compression loading. The unaged NBR specimens were also prepared for comparison. In the thermal oxidative aging testing, NBR specimens were exposed to thermal air (short-time heating) in order to obtain the natural aging effect of NBR. The spacing between specimens was kept at 10 mm in the aging test chamber. The air around the specimens was kept in constant circulation during the experiment process. In the oil aging testing, 15# aviation hydraulic oil was selected as the experimental medium to simulate the working condition of NBR materials. The remaining experimental factors were consistent with the thermal oxidative aging tests.

### 2.3. Hardness Testing

The shore hardness tester was used to measure the shore hardness of NBR specimens (unaged and aged) before compression testing (according to GB/T 531.1–2008 [48]). The specimens were gently placed on the test bench. The needle of the shore hardness tester was pressed vertically into the surface of the specimen. The shore hardness was read within a second. Each NBR specimen was measured 10 times and the final results were averaged.

### 2.4. Compression Testing

Compression testing of NBR specimens was carried out by material testing machine (Instron–5967, Instron, Boston, MA, USA). NBR cylindrical specimens were selected after aging testing in two kinds of conditions to obtain the elastic properties. Pre-compression (5 times per group) was carried out before formal compression tests to reduce the testing errors. The surface of the cylindrical specimen was coated with a certain amount of talcum powder before formal tests to reduce the adhesion between the specimens and the fixture. The compression speed is 10 mm/min (according to GB/T 7757–2009 [49]).

### 2.5. Friction Testing

**Figure 3:**
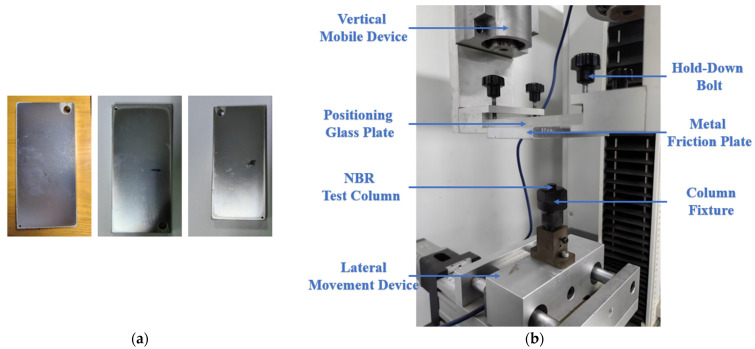
(**a**) The metal friction plates for friction testing. (**b**) The two-dimensional friction test bench for friction testing between NBR cylindrical specimens and metal friction plates.

Seals were prepared by directly contacting NBR material with the shaft or groove in the sealing structure. Contact pressure and shear stress are important factors in the sealing performance of NBR seals. The aging behaviors of NBR materials also affect the tribological behaviors of seals. This test was carried out to obtain the tribological behavior laws in this process. Figure 3a shows the metal friction plates (length is 13.0 ± 0.5 mm, width is 6.0 ± 0.5 mm, height is 1.0 ± 0.1 mm) with three kinds of surface roughness (0.6, 1.2 and 1.8) and they were selected for friction tests. Figure 3b shows the friction test bench which was used for observing and analyzing the tribological behaviors of the NBR seals and the metal structure. Detailed in Section 2.2, the NBR cylindrical specimens, taken after aging testing, were used for friction testing. The seal and its ancillary structures are always in an oil medium during the working process. Therefore, a lubricant film was pre-established between the friction contact pairs in the experiments based on this operating characteristic. The compression of specimens was controlled by changing the height of the metal friction plates. In the operation, the height of the metal friction plate was first slowly lowered until its surface gently fit the upper surface of the NBR cylindrical specimen, as shown in Figure 3b. And the indication of the sensor was synchronously observed to ensure that the lifting pairs were in a state of critical contact with each other. Subsequently, the operating software was operated to slowly lower the height of the friction plate. The compression rate of the cylindrical specimen was set to 10%. Finally, the reciprocal wear test was conducted with the aid of the program at room temperature (25 °C). The movement period was set to 1500 at 500 mm/min and movement distance was set to 10 mm.

### 2.6. Finite Element Model of Seals

#### 2.6.1. Material Constitutive Model

NBR is a kind of material with an obvious degree of nonlinearity. The nonlinear behavior of the material can be described well by the Mooney–Rivlin model [50,51,52,53]. Its parameters have excellent physical interpretability. It can also reflect the nonlinear strain capacity of the material intuitively. Therefore, the Mooney–Rivlin model was selected in the simulation to characterize the stress–strain relationship of NBR. The Mooney–Rivlin model can be expressed by Equation (1):

(1) $$W=C_{10}\left(I_{1}-3\right)+C_{01}\left(I_{2}-3\right)$$

where W is strain energy density, C_10_ and C_01_ are two ratios of Mooney–Rivlin and I_1_ and I_2_ are deformation tensors.

C_10_ and C_01_, obtained from compression data of NBR in different testing conditions, are shown in Table 1 and Table 2.

#### 2.6.2. Meshing

**Figure 4:**
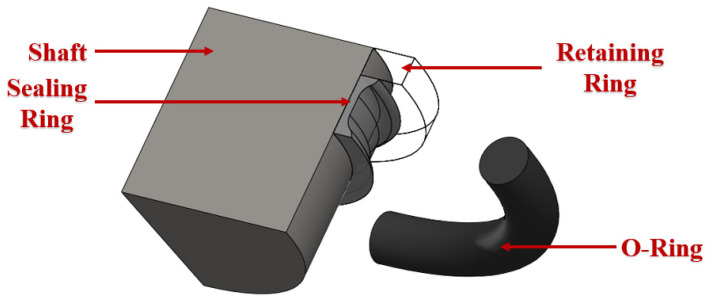
The sealing structure of NBR seals (quarter) (The three-dimensional model of seals).

A finite element model was established based on a combined sealing structure, as shown in Figure 4. The model was simplified to a certain extent according to the working characteristics of the combined seal structure. The combined seal structure is an axisymmetric structure. The constraints, boundary conditions and load conditions of the seal are all axisymmetric during the working process. The stresses, strains, displacements and deformations of the seals also have an axisymmetric distribution. Therefore, the finite element analysis and calculation were simplified into a two-dimensional axisymmetric model.

The element shape of the O-ring was quad-dominated and the structured element was selected. The O-ring was first drawn in the shape of a copper coin to obtain the regular shape of the grid cell. The element type was CAX4RH and hybrid formulation as well as reduced integration were selected. A total of 4800 elements were contained in the O-ring. The element shape of the sealing ring was quad-dominated and the free element was selected. The element type of the sealing ring was CAX4RH and 1755 elements were present. The element shape of the retaining ring was quad-dominated and the structured element was selected. The element type of the sealing ring was CAX4RH and 1260 elements were contained.

#### 2.6.3. Boundary and Load Condition

**Figure 5:**
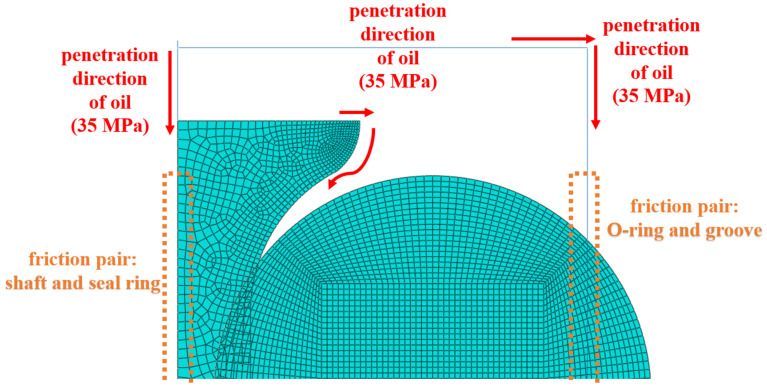
The pressure penetration method of the sealing finite element model.

The finite element analysis was divided into 3 main stages:

(1) The shaft was held stationary while the groove was displaced. The aim at this stage was to achieve a 13% compression of the O-ring for a pre-compression process.
(2) The displacement was further applied to the grooves until the assembly requirements were met for the subsequent application of oil pressure.
(3) The passage of aviation hydraulic fluid into the sealing system was simulated by pressure penetration. The pressure penetration method is shown in Figure 5. The load had to be increased gradually and the pressure was limited to the maximum value for all of the process. This was to avoid non-convergence in the step of loading.

The distribution of contact pressure is closely related to the sealing performance of the combined seal structure. In general, the effective sealing area is determined by the relationship between the contact pressure of the seal surface and the pressure of the working medium under service conditions. An effective seal is achieved when the contact pressure between the contact pairs is greater than the mass pressure. Otherwise, the contact pairs tend to separate which leads to seal failure due to excessive mass pressure in practical engineering situations. The method was still adopted in the further analysis to analyze the contact pressure distribution under various aging conditions.

## 3. Results and Discussion

### 3.1. Effect of Aging on Mechanical Performance

**Figure 6:**
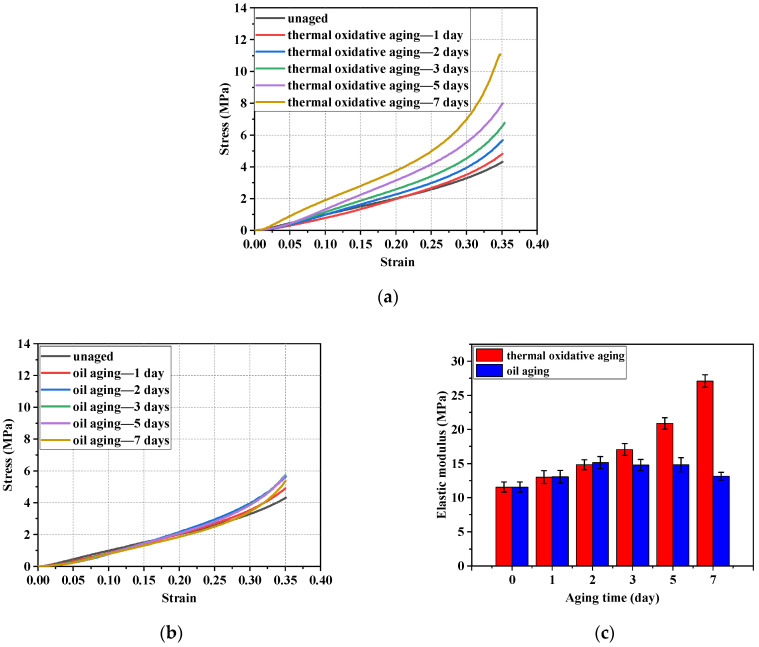
The compression curves and elastic modulus of the NBR specimens in different testing conditions. (**a**) Compression curves of NBR in thermal oxidative aging testing. (**b**) Compression curves of NBR in oil aging testing. (**c**) Elastic modulus of NBR with standard deviation in aging testing.

The compression curves and elastic modulus of the NBR specimens in different testing conditions were shown in Figure 6. It can be observed from Figure 6a,c that the elastic modulus increased with the increase in aging time in the thermal oxidative testing. The elastic modulus of NBR specimens aged for 7 days (27.10 MPa) was significantly larger than that of the unaged specimens (11.55 Mpa). It can be concluded from Figure 6b,c that, in oil aging testing, the elastic modulus was numerically close to that of the unaged NBR specimens, compared to that of the thermal oxidative aging testing. The elastic modulus of NBR specimens aged for 1 day (13.07 Mpa) and 7 days (13.15 Mpa) were relatively close in value and the difference between their values was 0.61%. And they were greater than those of the unaged specimens (11.55 Mpa). This indicates that the elastic modulus of NBR specimens increased first and then gradually decreased with the increase in aging time. The elastic modulus for 7 days of thermal oxidative aging increased by 135.45% compared to that of the unaged, and the elastic modulus of 7 days of oil aging increased by 15.03% compared to that of the unaged. The changes in the molecular structure of NBR affected the elastic modulus of the NBR in the aging testing [10,11,12,13]. NBR molecules rearranged themselves to form a more compact structure in the thermal oxidative aging testing, which led to an increase in the elastic modulus [12]. However, the contact and reaction between the NBR and oxygen were inhibited by the presence of oil in the oil aging testing. In summary, the elastic modulus of NBR showed different trends in the two different aging tests.

**Figure 7:**
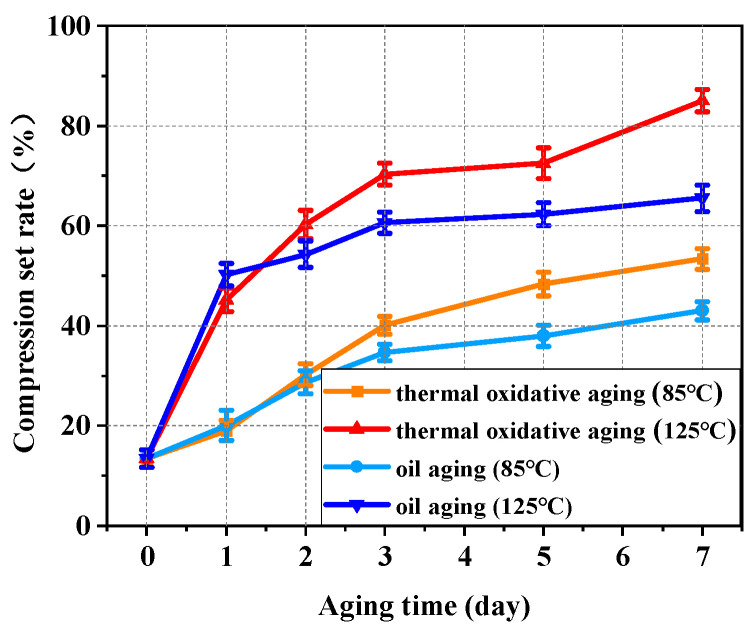
The compression set rate of NBR at 85 °C and 125 °C at different aging days.

The results of the compression set testing at 85 °C and 125 °C are shown in Figure 7. It can be concluded from the results that the compression set rate of NBR specimens at both service temperatures increased significantly with the increase in aging time. Compared with the elastic modulus of rubber, the compression set rate characterizes the elasticity of NBR and the recovery performance of NBR under different service conditions. The degree of compression set was greater at higher temperatures. Therefore, it was more difficult for NBR to recover its original elasticity at a higher working temperature in the testing. At 85 °C, the compression set rate for 7 days of thermal oxidative aging increased by 296.66% compared to that of the unaged, and the compression set rate for 7 days of oil aging increased by 219.39% compared to that of the unaged. At 125 °C, the compression set rate for 7 days of thermal oxidative aging increased by 531.65% compared to that of the unaged, and the compression set rate for 7 days of oil aging increased by 387.07% compared to that of the unaged. At 125 °C, NBR in the thermal oxidative environment showed the highest compression set rate of 85.02% on the 7th day. The compression set rate in the thermal oil environment at the same temperature was 65.56% on the 7th day. The oxidation was isolated by the oil environment. However, the compression set rate of the unaged group was only 13.46% and the adverse effects of aging on the NBR could not be avoided by the oil environment.

### 3.2. Effect of Aging on Tribological Behavior

**Figure 8:**
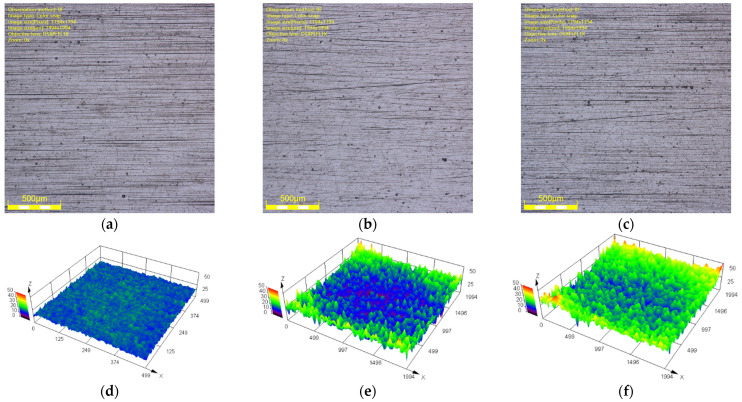
The initial surface morphology of metal friction plates. (**a**) The 2D morphology (roughness is 0.6). (**b**) The 2D morphology (roughness is 1.2). (**c**) The 2D morphology (roughness is 1.8). (**d**) The 3D morphology (roughness is 0.6). (**e**) The 3D morphology (roughness is 1.2). (**f**) The 3D morphology (roughness is 1.8).

**Figure 9:**
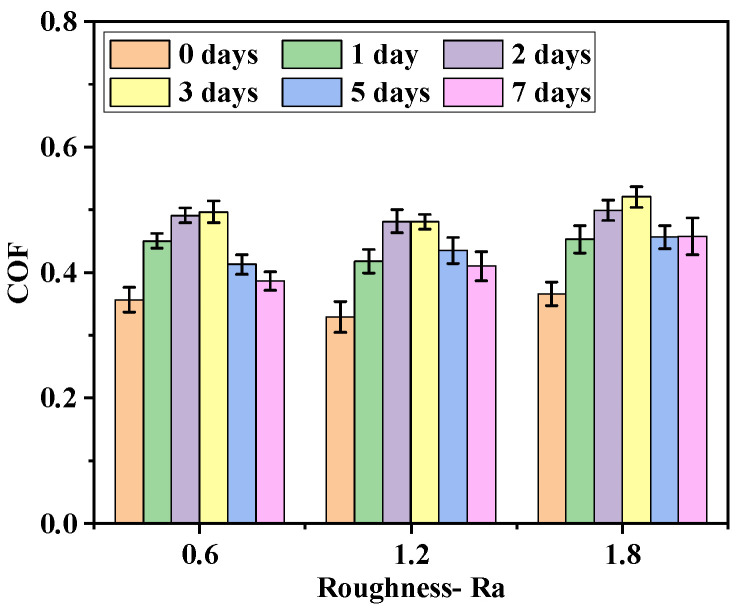
The variation in the COF with the roughness of metal friction plate at different aging days.

The initial surface morphology under optical digital microscope (DSX–510, OLYMPUS, Tokyo, Japan) is shown in Figure 8. The variation of the COF with the roughness of the metal friction plate at different aging days is shown in Figure 9. According to Figure 8 and Figure 9, the COF between the friction pairs did not easily increase as the roughness of the metal friction plate increased because of the combined effect of the adhesion and aging. When the roughness of metal plate was low, the distance between the rubber molecules and the metal atoms at the contact surface was close. The adhesion force was large, resulting in a large force and COF between friction pairs at this time. However, the adhesion gradually decreased as the roughness of the metal friction plate increased due to the increase of tiny undulations on its surface. The rubber surface became embedded in the microscopic grooves of the metal surface in the friction testing due to the increase in roughness, resulting in the enhancing of the plowing effect and increase in the COF.

**Figure 10:**
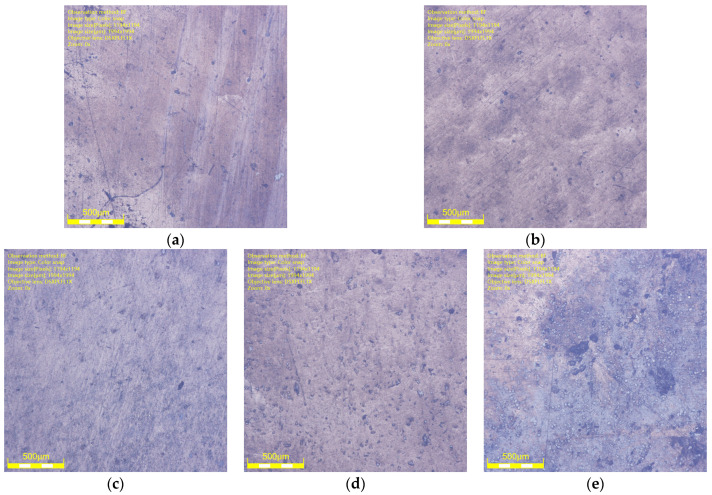
The surface morphology (2D) of NBR specimens at different aging days. (**a**) Aged for 1 day. (**b**) Aged for 2 days. (**c**) Aged for 3 days. (**d**) Aged for 5 days. (**e**) Aged for 7 days.

**Figure 11:**
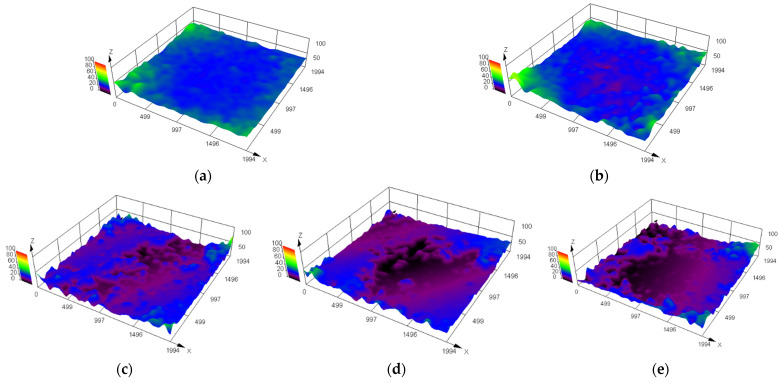
The surface morphology (3D) of NBR specimens at different aging days. (**a**) Aged for 1 day. (**b**) Aged for 2 days. (**c**) Aged for 3 days. (**d**) Aged for 5 days. (**e**) Aged for 7 days.

The surface morphology of NBR specimens under an optical digital microscope at different aging days is shown in Figure 10 and Figure 11. It can be seen that the rubber surface was destroyed by oil aging. The state of contact and friction between the rubber surface and the metal surface were affected. The defects and impurities on the rubber surface increased as the aging time increased. For example, the surface of NBR specimens aged for 1 day was flat and smooth. The surface height was uniform (blue area in Figure 11). However, the surface of NBR specimens aged for 2 days gradually developed some surface defects (reddish–brown area in Figure 11). The surface defects became increasingly dense and intense with the increase in aging time. It was detrimental to the working surfaces of NBR seals.

**Figure 12:**
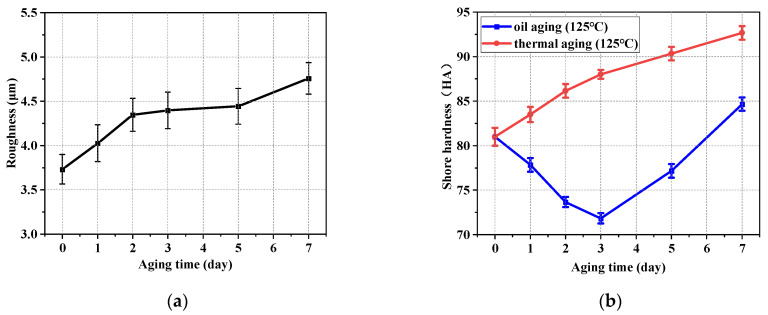
Variation in roughness and shore hardness of NBR working surface with aging time. (**a**) Variation in roughness with aging time at 125 °C. (**b**) Variation in shore hardness with aging time at 125 °C.

Figure 12 shows the variation in Sa (a roughness evaluation parameter based on area morphology) and the shore hardness of rubber specimens with aging time. It can be observed from Figure 12a that the working surface condition of NBR was gradually changed by oil aging, resulting in a rougher working surface. The trend in Sa is validated by comparison with the change in surface morphology (as shown in Figure 10 and Figure 11). It can be observed from Figure 12b (the red curve) that the variation of trends in shore hardness increased with the increase in aging time in the thermal oxidative aging testing. The increase in the hardness meant that the working surface of the NBR specimen was more resistant to deformation. The increase in the hardness also made the NBR specimen more easily keep its shape and size stable. However, the shore hardness of NBR decreased first and then increased as the aging testing proceeded in the oil aging testing (the blue curve). The trend in hardness changed on the 3rd day of aging. The comparison between the two curves illustrates that the swelling effect of NBR together with the aging behavior changed its hardness in the oil aging testing. The fact that the hardness can be decreased by the swelling effect of NBR was proved by comparison, as seen in Figure 12b. There is an interaction between the NBR and the oil, resulting in the expansion of the volume of NBR and a change in the physical and mechanical properties. The connections between the NBR molecules are broken by oil molecules and the structure of the rubber becomes less compact, resulting in a reduction in the hardness. In the early stage of aging (Day 0–Day 3), the aging effect was relatively weak. The swelling effect between NBR and the medium made the hardness of NBR gradually decrease at this time. As the aging effect increased and the swelling was gradually saturated (Day 3–Day 7), the aging effect dominated and the hardness of NBR increased.

**Figure 13:**
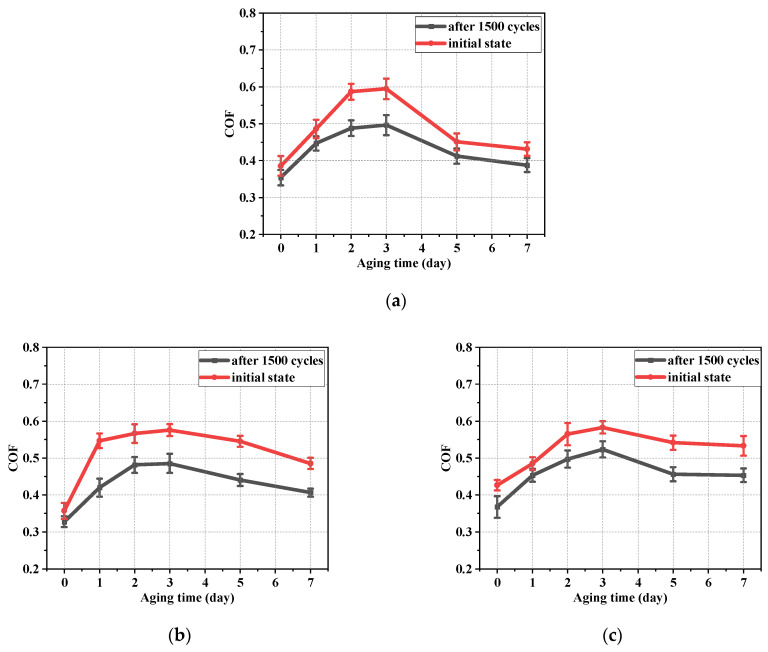
The variation in the COF between NBR and metal friction plates with aging time. (**a**) The roughness of the metal friction plate is 0.6; (**b**) the roughness of the metal friction plate is 1.2; (**c**) the roughness of the metal friction plate is 1.8.

The variation in the COF between NBR and metal friction plates with aging time is shown in Figure 13. It can be observed that the COF between friction pairs increased first and then decreased with the increase in aging time. The COF, after a certain number of cyclic friction tests, was significantly lower than that of the initial state. This trend was explained by using the shore hardness trend in the NBR specimen during the aging testing in Figure 12b. NBR is a kind of superelastic material. It contacted the working surface of the metal more fully when the hardness was lower (Day 0–Day 3) compared to in the unaged condition. At the same time, the increase in the contact area was also conducive to the action of the hysteresis force. The contact between the NBR and the metal was stronger due to the above factors, resulting in a gradual increase in the measured friction force and the COF. Therefore, the COF exhibited an increasing monotonic trend in the early and middle stages of aging (Day 0–Day 3). However, the ability of the NBR surface to resist deformation became stronger due to the gradual increase in the shore hardness (Day 3–Day 7) of the NBR surface. The contact area was gradually reduced between the friction pairs in this period. The friction force and COF measured in this period gradually decreased.

### 3.3. Effect of Aging on Sealing Characteristics

#### 3.3.1. Contact Pressure

**Figure 14:**
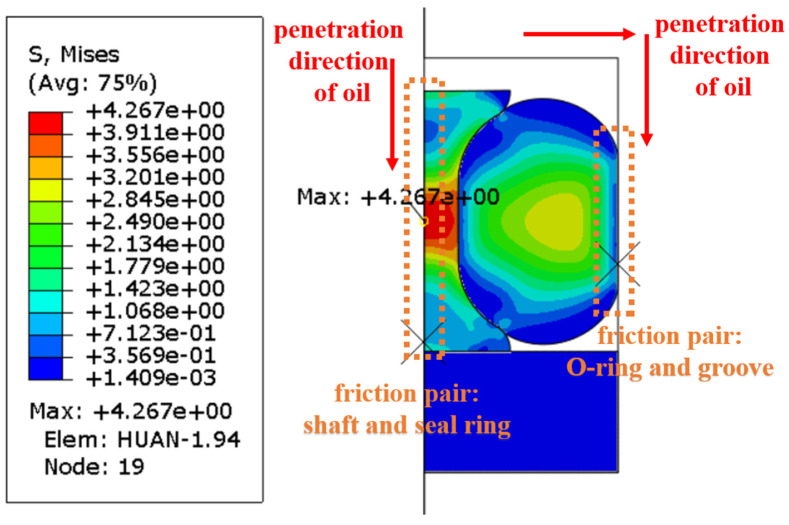
Schematic diagram of seals and analysis methods.

During the operation of a combined seal structure, the distribution of the contact pressure between important friction pairs is critical to the sealing performance. According to the penetration direction of hydraulic oil, the friction pairs (the shaft and the seal ring; the O-ring and the groove structure) were focused on the process of finite element analysis, as shown in Figure 14.

**Figure 15:**
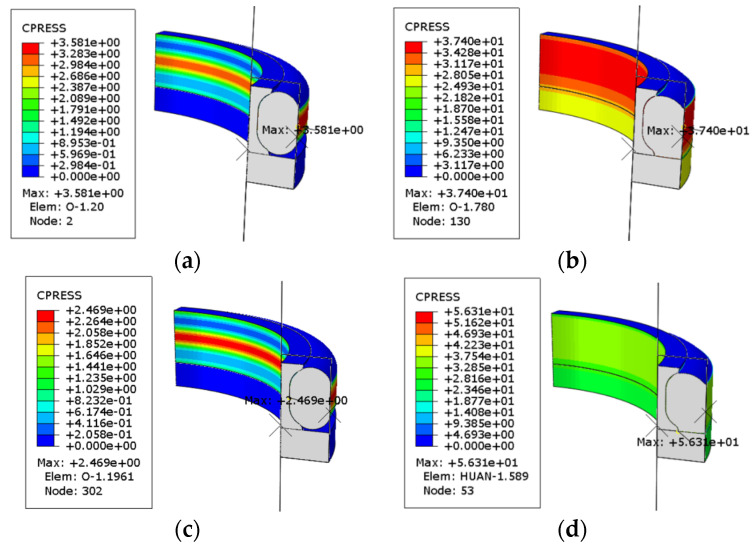

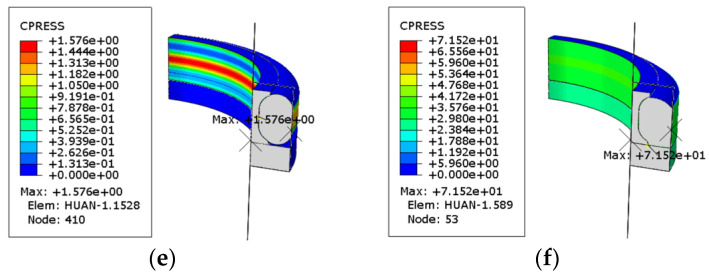
Contact pressure distribution of NBR seals in different testing conditions. (**a**) Unaged (before oiling); (**b**) unaged (after oiling); (**c**) thermal oxidative aging for 3 days (before oiling); (**d**) thermal oxidative aging for 3 days (after oiling); (**e**) oil aging for 3 days (before oiling); (**f**) oil aging for 3 days (after oiling).

Figure 15 shows the contact pressure distribution before and after oiling in different testing conditions. It can be seen that the value of the maximum contact pressure increased significantly after oiling and it increased more after aging testing. The forces on the O-ring were mainly derived from the compression and friction of the other seals and the outer groove structure before oiling. Its stress map in the axial direction was relatively regular at this time. However, the O-ring was forced along the direction of oil pressure deformation by the pressure of the oil medium penetration, resulting in a change in the location of the stress concentration of the seal structure.

**Figure 16:**
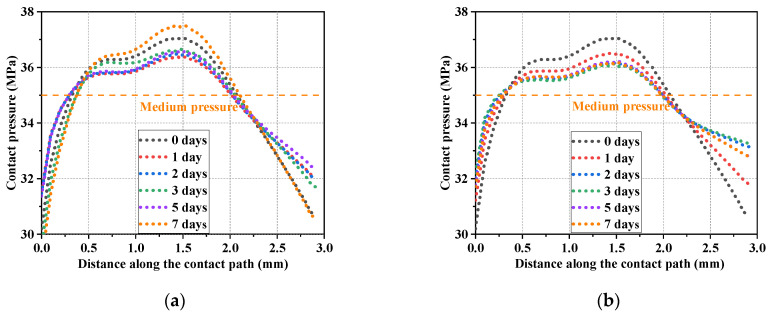
Contact pressure distribution along the contact path (shaft and seal ring). (**a**) Thermal oxidative aging group; (**b**) oil aging group.

Figure 16 shows the distribution of contact pressure between the shaft and seal ring along the contact path in different testing conditions. The distribution curve of the contact pressure was divided into three intervals along the penetration direction of the medium. In the first interval, the contact pressure gradually increased under the external force of fluid pressure until it equaled the value of the medium pressure. The contact pressure showed an essentially monotonic numerical increase in this interval and the trend was stable. In the second interval, with the penetration of the medium and interaction between the seals, the contact pressure continued to increase and was already greater than the medium pressure. In this interval, the contact pressure increased first and then decreased. In the third interval, the penetration of the medium was gradually saturated. The contact pressure decreased until the seal came into contact with the lower retaining ring.

**Figure 17:**
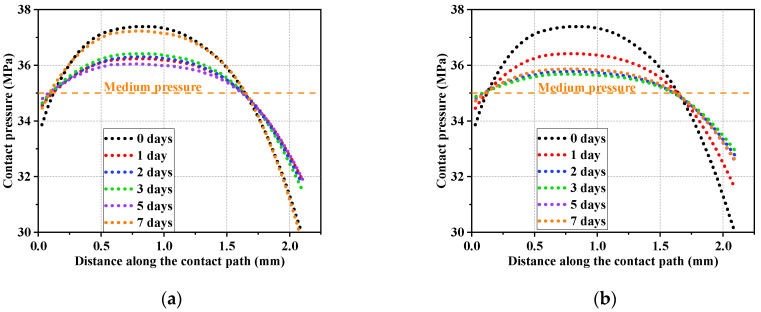
Contact pressure distribution along the contact path (O-ring and groove). (**a**) Thermal oxidative aging group; (**b**) oil aging group.

Figure 17 shows the distribution of contact pressure between the O-ring and the groove structure along the contact path in different testing conditions. It can be observed from the results that the contact pressure showed a monotonic trend in the first and third intervals. The trend in the contact pressure in the second interval resembled a parabola with a downward opening. The fluctuation in the value was smaller compared to the previous friction pair (the shaft and the seal ring), due to the fact that there were fewer interacting structural elements in the friction pair between the O-ring and groove.

**Figure 18:**
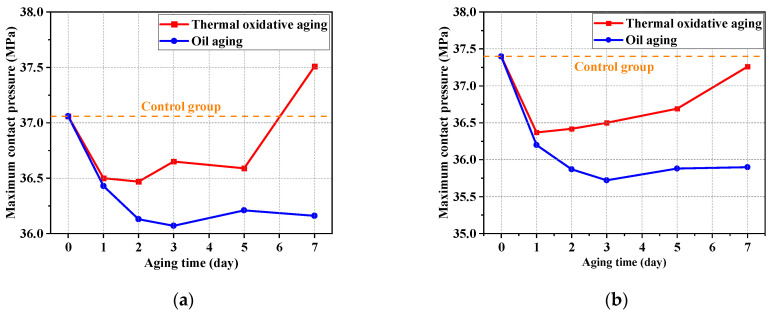
(**a**) Variation in maximum contact pressure in aging testing (the sealing friction pair between the shaft and seal ring). (**b**) Variation in maximum contact pressure in aging testing (the sealing friction pair between the O-ring and groove).

Figure 18a shows the variation in maximum contact pressure between the shaft and seal ring in aging testing. It can be seen from the results that the maximum contact pressure in thermal oxidative aging was always larger than that in oil aging at the same aging time. The difference in maximum contact pressure between the two kinds of aging testing was greatest in Day 7 and it reached 1.35 MPa. The maximum contact pressure did not vary regularly with aging time. Figure 18b shows the variation in maximum contact pressure between the O-ring and groove in aging testing. It can be seen from the results that the maximum contact pressure in thermal oxidative aging was also always larger than that in oil aging for the same aging time. It also can be observed that the value in this friction pair decreased first and then increased as the aging progressed. They were all smaller than that of the unaged (37.40 MPa). Between the shaft and seal ring, the maximum contact pressure of 7 days of thermal oxidative aging increased by 1.21% compared to the that of the unaged, and the maximum contact pressure for 7 days of oil aging decreased by 2.43% compared to the unaged value. Between the O-ring and groove, the maximum contact pressure of 7 days of thermal oxidative aging decreased by 0.37% compared to the unaged value, and the maximum contact pressure of 7 days of oil aging decreased by 4.01% compared to the unaged value.

#### 3.3.2. Proportion of Effective Sealing Area

**Figure 19:**
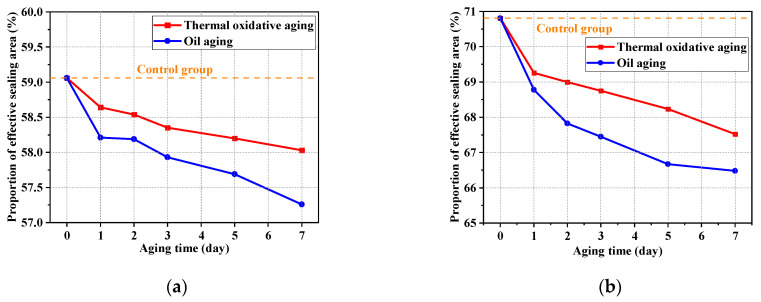
Variation in the proportion of effective sealing area in aging testing. (**a**) The sealing friction pair between the shaft and seal ring; (**b**) the sealing friction pair between the O-ring and groove.

Figure 19 shows the proportion of effective sealing area in aging testing. The seal is effective between friction pairs when the contact pressure is larger than the medium pressure. The case of equal values is a critical state. The second intervals in Figure 16 and Figure 17 were used to calculate the proportion of effective sealing area. It can be observed that the proportions of effective sealing area in oil aging were lower than those in thermal oxidative aging. The proportion of effective sealing area decreased gradually in both conditions. The contact area between friction pairs was reduced and the sealing performance was deteriorated by oil aging. Between the shaft and seal ring, the proportion of effective sealing area for the 7 days of oil aging decreased by 3.05% compared to that of the unaged. Between the O-ring and groove, the proportion of effective sealing area for 7 days of oil aging decreased by 6.11% compared to the unaged value.

**Figure 20:**
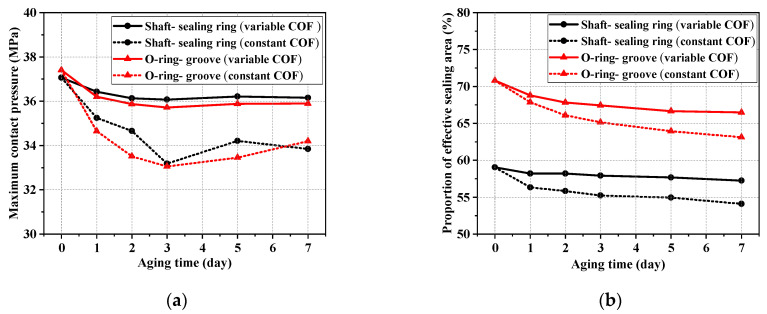
The comparative variation in the maximum contact pressure and proportion of effective sealing area, considering whether or not changes in the COF in oil aging testing are taken into account. (**a**) The maximum contact pressure in two important sealing friction pairs. (**b**) The proportion of effective sealing area in two important sealing friction pairs.

Figure 20 shows the comparative variation in maximum contact pressure and the proportion of effective sealing area. The influence of the COF change was compared and analyzed. It can be seen that the maximum contact pressure in the friction pairs was lower when the COF was constant. This indicates that the structures are inaccurate and unreliable if they are designed for lower contact pressure (conditions of constant COF). It can also be seen that the proportions of effective sealing area were even lower in the conditions of a constant COF. Therefore, the change in the COF should be considered with the aging time because the tribological behaviors of NBR were also affected by the influence of oil and thermal aging.

## 4. Conclusions

In summary, this study focused on the variation in seal characteristics through a finite element model of seals, with the method of coupling the mechanical behavioral changes with the tribological behavioral changes of NBR in oil and thermal environments. According to the results and the analysis above, we have the following points:

(1) The elastic modulus increased with the increase in aging time in thermal oxidative aging. The elastic modulus for 7 days of thermal oxidative aging increased by 135.45% compared to the unaged value, and the elastic modulus for 7 days of oil aging increased by 15.03% compared to the unaged value. The compression set rate of NBR increased significantly with the increase in aging time and aging temperature. It was difficult for NBR to regain its original elasticity under a high ambient temperature.
(2) Oil aging gradually changed the working surface condition of NBR and resulted in a rougher working surface. The COF between sealing friction pairs increased in the first 3 aging days and then decreased in other aging days. Moreover, after a certain number of friction cycles, the COF was significantly lower than that of the initial state.
(3) The maximum contact pressure decreased by 2.43% between the shaft and sealing ring and decreased by 4.01% between the O-ring and groove. The proportion of effective sealing area decreased by 3.05% between the shaft and sealing ring and decreased by 6.11% between the O-ring and groove. Oil aging decreased the maximum contact pressure and proportion of effective sealing area compared to the unaged case. The sealing characteristics of NBR seals worsened with the increase in aging time. In addition, the proportion of effective sealing area between the O-ring and groove structure in general were greater than that between the shaft and seal ring. It showed better sealing characteristics between the O-ring and groove.

## Figures and Tables

**Table 1:** C_10_ and C_01_ in thermal oxidative aging testing.

	0 Days	1 Day	2 Days	3 Days	5 Days	7 Days
C_10_	1.0567	−0.8381	−1.2246	−1.6025	−3.0367	−2.5138
C_01_	0.1658	1.5967	2.0287	2.4740	3.8295	3.8574

**Table 2:** C_10_ and C_01_ in oil aging testing.

	0 Days	1 Day	2 Days	3 Days	5 Days	7 Days
C_10_	1.0567	−0.6055	−2.0126	−2.1378	−1.7491	−1.4055
C_01_	0.1658	1.4429	2.5909	2.6745	2.3664	2.0035

## Data Availability

Data can be obtained from the authors on request.
